# A 5-emotions stimuli set for emotion perception research with full-body dance movements

**DOI:** 10.1038/s41598-023-33656-4

**Published:** 2023-05-30

**Authors:** Julia F. Christensen, Laura Bruhn, Eva-Madeleine Schmidt, Nasimeh Bahmanian, Sina H. N. Yazdi, Fahima Farahi, Luisa Sancho-Escanero, Winfried Menninghaus

**Affiliations:** 1 Department of Language and Literature, Max-Planck-Institute for Empirical Aesthetics, Frankfurt/M, Germany; 2 Department of Cognitive Neuropsychology, Max Planck Institute for Empirical Aesthetics, Frankfurt/M, Germany; 3 Max Planck School of Cognition, Max Planck Institute, Leipzig, Germany; 4 Department of Modern Languages, Goethe University, Frankfurt, Germany; 5 3Fish Corporate Filmmaking, Istanbul, Turkey; 6 Pfalztheater Kaiserslautern Dance Company, Kaiserslautern, Germany

**Keywords:** Neuroscience, Psychology

## Abstract

Ekman famously contended that there are different channels of emotional expression (face, voice, body), and that emotion recognition ability confers an adaptive advantage to the individual. Yet, still today, much emotion perception research is focussed on emotion recognition from the face, and few validated emotionally expressive full-body stimuli sets are available. Based on research on emotional speech perception, we created a new, highly controlled full-body stimuli set. We used the same-sequence approach, and not emotional actions (e.g., jumping of joy, recoiling in fear): One professional dancer danced 30 sequences of (dance) movements five times each, expressing joy, anger, fear, sadness or a neutral state, one at each repetition. We outline the creation of a total of 150, 6-s-long such video stimuli, that show the dancer as a white silhouette on a black background. Ratings from 90 participants (emotion recognition, aesthetic judgment) showed that intended emotion was recognized above chance (chance: 20%; joy: 45%, anger: 48%, fear: 37%, sadness: 50%, neutral state: 51%), and that aesthetic judgment was sensitive to the intended emotion (beauty ratings: joy > anger > fear > neutral state, and sad > fear > neutral state). The stimuli set, normative values and code are available for download.

## Introduction

### Summary

Much emotion perception research has focussed on emotion recognition from the face^1–8^. However, several studies have shown that emotional expressions for faces and bodies are not always aligned^9–11^, and that interindividual differences modulate emotion recognition for faces versus bodies^2,10^. Therefore, there have been calls for more research into emotion recognition competence for full-body movements, both in clinical and in non-clinical settings^12–18^. And refined tests that measure individual differences in emotion recognition ability objectively, and which do not rely on self-report, are of broad interest. The usefulness of such tests to measure emotion recognition ability hinges on suitable stimuli materials.

We here present a novel type of full-body stimuli for experimental psychology of emotion: expressive dance movements. We created a stimulus set comprising 150 6-s long, high-quality videos of one dancer performing sequences of full-body movements (30 sequences of choreographed Western contemporary and ballet dance). The dancer repeated each of these 30 sequences five times each, with one of five different emotional intentions at each repetition (joy, anger, fear, sadness, and one neutral state; 30 sequences x five emotions = 150 stimuli). A validation experiment with a normative sample of N = 90 participants showed that the intended emotional expression of the dancer was recognized above chance in 139 of these stimuli. The stimuli set is open access and includes normative emotion recognition rates and subjective value judgments (aesthetic and emotional intensity ratings) for each stimulus. As we outline at the end of “Background literature” section, one novelty of the stimuli set is that the stimuli can be used both for *explicit* emotion recognition tasks (e.g., for forced-choice emotion recognition paradigms), as well as for *implicit* emotion recognition tasks (e.g., a distractor rating task that implicitly measures the sensitivity of the individual to the different emotion categories of the stimuli).

### Background literature

Emotion recognition accuracy is commonly assessed by means of perceptual tasks where participants are asked to decode or guess the emotional intention of other people on stimuli showing faces, bodies, situations, stories, music, etc. (e.g., the Multi-Factor Emotional Intelligence Scale (MEIS)^19^ or the Diagnostic Analysis of Nonverbal Accuracy (DANVA2)^20^). ‘Accurate’ emotion recognition on these tasks refers to an objective test. A normative sample of participants is asked to guess the emotion intended by a person acting as expressor in the stimuli (e.g., through facial or bodily expression of emotion). For example, if the intended emotion by the expressor is “anger” and “anger” is guessed above chance by participants, then “anger” is taken as the ‘correct’ response for this stimulus, or, in other words, this stimulus “works”. Stimuli, where the recognition rate of the intended emotion by the expressor is below chance in a normative sample should be discarded from a stimulus set, as this would be evidence that the stimulus does not “work”. Subsequently, if a participant in a new experiment does not guess a stimulus as “anger” that was (a) intended by the expressor to express anger, and (b) was recognized as such above chance by a normative sample, their answer is, in consequence, defined as ‘wrong’. A single person’s emotion recognition accuracy across all stimuli can now be compared against the emotion recognition accuracy of the normative sample.

For example, the Geneva Emotion Recognition Test Short (GERT-S)^21^ comprises 42 video stimuli showing the upper body and face of actors expressing 14 different emotions with their facial expression while saying a nonsensical sentence with different emotional intonations. Similarly, the Emotion Recognition Index (ERI) measures emotion recognition accuracy for four emotions in face and voice stimuli^6^. It is based on the picture stimuli set by Ekman and Friesen^22^, and on voice recordings from a published corpus^23^.

Full-body emotion recognition research has, so far, to a large extent, relied on video stimuli of ‘emotional actions’ (e.g., bending the head down in sadness, clenching a fist in anger, jumping for joy, recoiling in fear, etc.). Available full-body emotion stimuli likely measure the cognitive *recognition* of emotional *actions*, rather than the sensitivity to the kinematics of emotional intentions in full-body movements, as discussed in previous work^24,25^. Besides, emotions are not always expressed as specific full-fleshed emotional actions (e.g., bending the head down in sadness, clenching a fist in anger, jumping for joy, recoiling in fear, etc.). Especially in the first stages of the development of an emotion, these are rather implied within subtle kinematics of an individual’s movements; a person can wave angrily, happily, sadly, etc. And, the ability to detect these subtle kinematic differences in full-body movements could be argued to be *genuine* emotion recognition ability.

A new line of research, therefore, focusses on requiring participants to recognise emotions from stimuli showing individuals performing the *same* simple transitive movements—walking or throwing—across different emotional intentions (e.g., joy, sad, fearful, angry, and a neutral state)^26–30^. Expanding this approach, we propose that it is possible to generate phrases of more complex full-body movements or full-body gestures. Choreographed sequences of dance movements afford exactly this. Dance is, in its essence, a kind of human expressive body movement^31^. And, professional dancers are ideal models for the creation of dance stimuli materials in emotion science because they are trained to express different emotional intentions, with one and the same dance movement^32–34^. Subtle variations in how a dancer performs a dance movement with different emotional intentions conveys these intentions to observers^35–37^.

This phenomenon is comparable to language, where a single sentence can be pronounced with different emotional qualities (intonation) (e.g., angry or happy), depending on how the expressor modulates their voice with their breathing and the muscles of their vocal tract. For instance, stimuli for the *Multimodal Emotion Recognition Test* (MERT)^38^, and the *Test for Emotion Recognition from the Singing Voice*^39^ were created with actors and singers that either spoke or sang a pseudo-linguistic sentence (“*Ne-Kalibam-Sut-Molaine*”) at several repetitions with different emotional intentions. Computational analyses of the physical speech contours of these utterings revealed that these voice stimuli vary according to specific physical parameters of the sound. These parameters are picked up by human listeners and the intended emotions accurately decoded^40–42^.

The Warburg Dance Movement Library (WADAMO Library)^32^ was the first movement stimulus library that was created following this rationale from the research on the perception of emotional speech, but with dance movements. It contains 245 6-s-long video clips of Western ballet and contemporary dance of two different expressive categories. Four dancers were instructed to perform several short dance choreographies of eight counts twice, once *with*, and once *without* emotional expressivity. Across several experiments, participants without dance experience accurately identified the dancers’ intended emotional expressivity (expressive *versus* neutral state, i.e., no expressivity)^32,43^. The McNorm dance movement library^44^ was the first library to contain five different emotional expressions for each dance movement sequence. One dancer performed Western contemporary dance movement sequences five times, with a different emotional expressive intention at each repetition (joy, sad, angry, fearful, and a neutral state). The neutral category consisted of the same movements, technically correct, but without any emotional expressivity. This latter neutral category is comparable to the “inexpressive” category of the WADAMO library, and to the “neutral” emotion stimuli category of all stimulus corpora since Ekman and Friesen^22,45^ (e.g., Atkinson et al.^46^). The McNorm library contained 73 video stimuli of varying lengths (6.6–42.8 s) and stimuli were rendered as point lights to maximally reduce visual information about the dancer. Average emotion recognition by participants was 48.96%.

Importantly, in addition to serving in an explicit emotion recognition task, the WADAMO library was also used to assess individuals’ sensitivity to the emotional expressiveness *implicitly*. Namely, different groups of participants were asked to make simple aesthetic judgments about the video clips (i.e., liking and beauty judgments). Participants systematically liked videos intended to be expressive more and found them more beautiful. Orlandi and colleagues (2020) used a similar approach, contrasting observers’ aesthetic judgment to emotionally expressive and inexpressive dance movement sequences^47^. Also here, participants rated the videos intended to be expressive as more beautiful than the inexpressive versions of the same sequences. The results of these experiments^32,43,47^ form the basis for the idea that dance movement stimuli could be used to assess emotion recognition accuracy *implicitly*. If observers—who are unaware of the intended emotional expressivity (i.e., they have not been told about the different intentions, like in an explicit emotion recognition task)—systematically provide higher aesthetic judgments (e.g., beauty or liking ratings) for expressive than for inexpressive versions of the *same* sequence, then the aesthetic judgment is an implicit measure of the person’s sensitivity to the intended expressivity in the movement.

### Objectives

The objectives of this project were, first, to create a new stimulus set with a high level of experimental control. Dance movement sequences and visual characteristics of the stimuli were controlled, and stimuli length was equalized as much as possible to 6 s. Second, we set out to provide normative values of emotion recognition and aesthetic judgment for all created stimuli. Third, we identified the stimuli with highest emotion recognition rates and that were recognized above chance to provide a stimuli table with all values for future stimuli selection. Fourth, we explored interindividual differences in emotion recognition and beauty ratings (personality traits and aesthetic responsiveness).

### The present study

We designed and created a new dance movement stimuli set based on the groundwork from previous stimulus creation procedures of dance stimuli sets^32,37,48–53^, which ensured requirements for experimental control^31,54^. During the subsequent norming experiment, 90 participants watched the stimuli (video clips of 6 s length), performed a forced-choice emotion recognition task and provided ratings for how beautiful and how intense they thought the stimuli were. A short video about the stimuli creation is available here: https://www.youtube.com/watch?v=Eij40jtw8WE.

**Figure 1:**
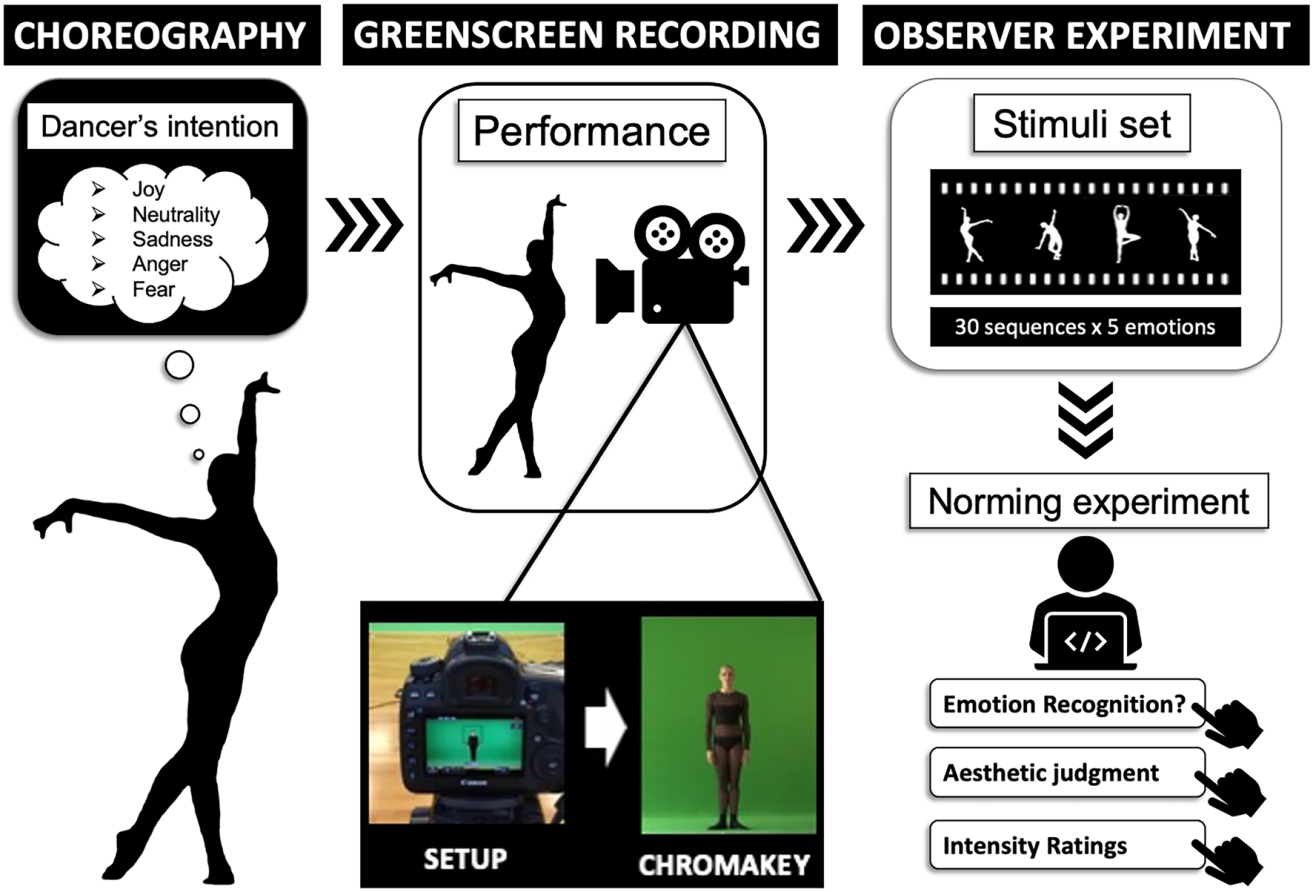
Stimuli Creation Procedure. The stimuli creation procedure was based on previous work^32,37,48–53^, and respected requirements of experimental control for dance stimulus materials^31,54^. Choreography of the 30 sequences (of Western contemporary and ballet dance) took place prior to the recording session and was led entirely by the dancer in conversation with two of the authors with professional dance experience (JFC and LSE). Filming of the dance sequences took place at the Max-Planck-Institute for Empirical Aesthetics in Frankfurt/M. For filming, a Canon EOS 5D Mark IV camera was used, with a Canon EF 24–105 mm f/4 L IS USM lens (settings: e.g., framerate (raw) at 50fps and framerate (output) at 25 fps. White balance: 5000 k, shutter speed: 1/100 s, and ISO: 400. The video format: H.264, aspect ratio: 16:9, and resolution: 1920 × 1080). A standard 6 × 3 m chroma-key greenscreen background was used to allow for the creation of additional visual preparations of the stimuli, such as silhouette videos and blurred faces. For this, dedo-stage lights (7 dedo heads, dimmers and stands kit) were required to illuminate the entire greenscreen and to minimise shadows. Postproduction was done using *Adobe After Effects* 2019 and *Adobe Premiere Pro* 2019. All footage was trimmed to the exact start and end points of the movements. Each clip was rendered into a separate file in an uncompressed format and the title was added, as specified verbally by the dancer during the recording. Before saving, the sound tracks (speech and ambience noise) of the clips were removed. Using *Adobe After Effects*, “Keylight” effect was added to all files, and the background removed from each clip. The “Level” effect (setting: output black = 255) was further applied to each clip to colour the extracted foregrounds white (the visible dancer silhouette). “Opacity” keyframes were then added to the beginning and the end of each clip to allow for a fade-in and fade-out of each clip (8 frames). Finally, each clip was rendered as a separate file in H264 format. The dancer was Ms Anne Jung and her informed consent for publication of identifying information, images and film in an online open-access publication were obtained. A short video of the creation process is available here: https://www.youtube.com/watch?v=Eij40jtw8WE.

See Fig. 1 for an illustration of the stimuli creation procedure and the norming experiment.

## Results

Emotion recognition was calculated for all 150 stimuli as an objective test. “Correct” emotion recognition was set to be when the participant had selected the emotion that the dancer intended while dancing (see also “Background literature” section). Emotion recognition accuracy for each emotion was obtained for each participant. All data and code are available on the OSF: https://osf.io/uecg9/?view_only=e5a5661b89104701aca750101325d30f.

### Preliminary data analyses

During stimuli creation, some sequences were performed more than once. These were cases, where the dancer was unsatisfied with her performance and asked to repeat the sequence. Therefore, the number of total stimuli was 173 stimuli (including 23 duplicates that were deleted once emotion recognition rates were obtained). The 173 stimuli were divided into three sets for three separate online experiments. Fifteen videos of the stimuli set were randomly selected and included in all three separate online norming experiments. To confirm that emotion recognition rates between the three sets of stimuli were equivalent, we performed comparative analyses. These showed equivalent emotion recognition rates and aesthetic judgment; hence, data from the three experiments was aggregated and duplicates were removed, based on the highest emotion recognition rate. These are set out in the supplementary materials (section 1).

### Emotion recognition accuracy

Data were non-normally distributed and non-parametric tests were performed.

**Figure 2:**
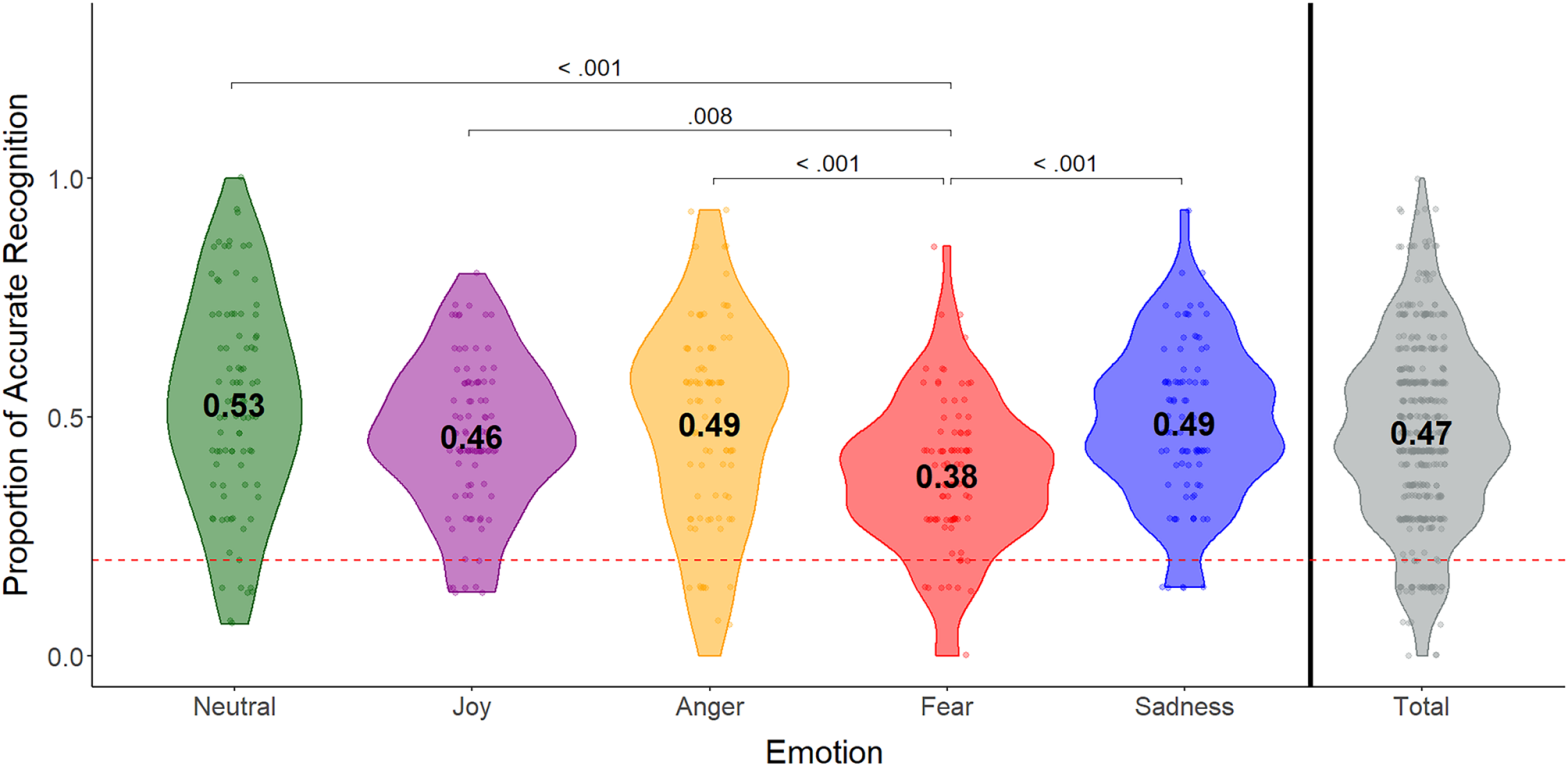
Emotion recognition accuracy across intended emotions. Mean and variability of emotion recognition accuracies for each emotion, based on participant-specific emotion recognition rates *p* values are Bonferroni-corrected. Dotted line illustrates chance level (100%/5 emotions = 20%)—all emotion categories were recognized above chance level on average. Stimuli expressing fear were recognized significantly less well than all other emotional categories, but still above chance.

A one-sample Wilcoxon signed rank test was used to determine whether emotion recognition accuracy was above chance. On average, participants recognised the emotion intended by the dancer in 46.8% of trials (± 19.04), significantly above the chance level of 20% (100/5 emotions = 20%), across all emotions (V = 97,137, *p* < .001, h = .579). The same was true for each emotion separately (i.e., participants recognised above chance level when the dancer expressed *joy* (V = 3891, *p* < .001, h = .565), *anger* (V = 3969, *p* < .001, h = .615), *fear* (V = 3662.5, *p* < .001, h = .397), *sadness* (V = 4080, *p* < .001, h = .62), and *neutral state* (V = 3950, *p* < .001, h = .698). See Table 1 and Fig. 2.

**Table 1:** Summary emotion recognition accuracy for each emotion.

Emotion	Average recognition rate (± *SD*) (%)	Median recognition rate (%)	Recognition greater than chance?	Statistical test
Joy	46.1 (± 15.79)	44.8	Yes	V = 3891, * p* < .001, h = .565
Anger	48.6 (± 21.39)	53.3	Yes	V = 3969, * p* * p* < .001, h = .615
Fear	37.8 (± 15.74)	37.9	Yes	V = 3662.5, * p* < .001, h = .397
Sadness	48.8 (± 16.33)	48.3	Yes	V = 4080, * p* < .001, h = .62
Neutral state	52.7 (± 21.98)	53.3	Yes	V = 3950, * p* < .001, h = .698
All emotions	46.8 (± 19.04)	46.7	Yes	V = 97,137, * p* < .001, h = .579

Average and median emotion recognition accuracies for N = 150 stimuli of each emotional intention rated by N = 90 participants (across three experiments with 30 participants each), and Wilcoxon signed rank tests to determine emotion recognition above chance level (100/5 = 20%). Data in this table are based on raw recognition values. Data is non-normally distributed.

Subsequently, a Friedman’s ANOVA was used to determine whether emotion recognition accuracy differed between stimuli of different categories of intended emotions (*joy, anger, fear, sadness, neutral state*) (χ^2^(4) = 31.61, *p* < .001). Wilcoxon signed rank tests with Bonferroni correction (significance level at .005) were used to follow up the significant main effect. Emotion recognition accuracy for stimuli expressing fear was significantly lower than for all other emotional categories (*joy* (V = 813.5, *p* = .007, h =  − .168), *anger* (V = 687.5, *p* < .001, h =  − .218), *sadness* (V = 649.5, *p* < .001, h =  − .223) and *neutral state* (V = 722.5, *p* < .001, h =  − .301)). There were no significant differences between stimuli of any of the other intended emotion categories (all *p*s > .391). See Fig. 2.

Besides, we explored emotion recognition accuracy across the different categories of intended emotions in terms of correct and mis-classifications. The highest confusions between emotions were: Stimuli intended to express *joy* were most often misclassified as neutral state, i.e., in 23.1% of trials (correct classifications: 49.2%), anger as joy in 24.2% of trials (correct classifications: 50.5%), fear as sadness in 25.5% of trials (correct classifications: 39.5%), sadness as neutral state in 23.8% of trials (correct classifications: 47.5%), and neutral state as sad in 20.4% of trials (correct classifications: 52.61%). See Table 2 for a confusion matrix.

**Table 2:** Confusion matrix for emotion recognition accuracy across intended emotions.

Intended emotion	Perceived emotion				
	Joy	Anger	Fear	Sadness	Neutral state
Joy	**45.03**	15.33	7.22	8.37	24.06
Anger	24.5	**48.31**	4.94	3.22	19.03
Fear	5.22	8.97	**37.05**	26.03	22.74
Sadness	5.73	4.07	17.61	**49.67**	22.92
Neutral State	11.18	4.73	9.77	22.65	**51.67**

Overview of the average emotion recognition accuracies of N = 150 stimuli: correct classifications of the intended emotion of the dancer by the participants on the diagonal in bold, and misclassifications in all other cells. Data in this table are based on raw recognition values.

### Intensity ratings

**Figure 3:**
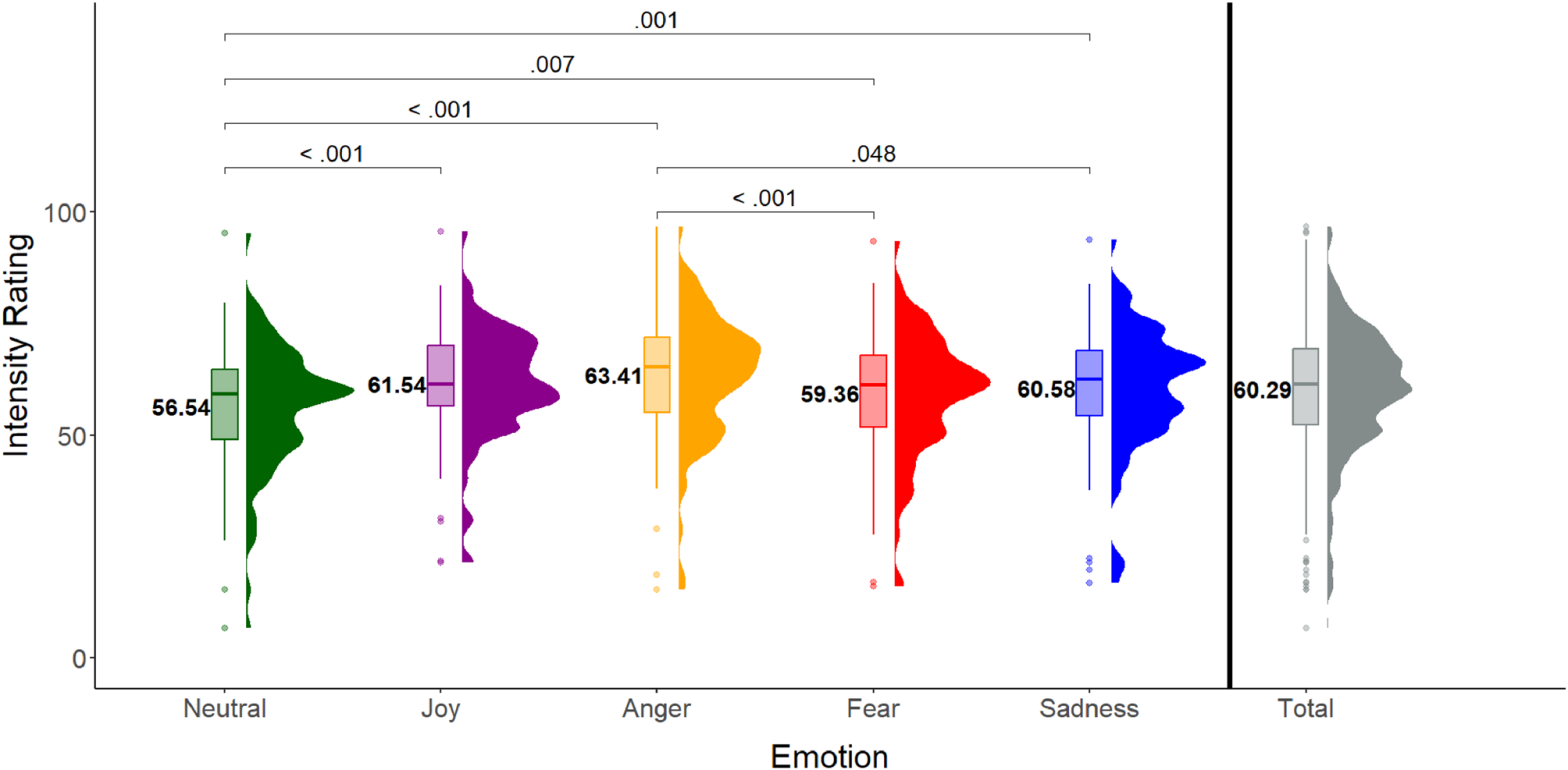
Average intensity ratings for 5 emotion categories. Mean and variability of Intensity ratings for stimuli of each emotion category as intended by the dancer and the total. *p* values are Bonferroni-corrected.

A Friedman’s ANOVA showed a main effect of Intended Emotion on participants’ Intensity ratings (χ^2^(4) = 48.49, *p* < .001), suggesting differences between categories of intended emotion. Follow-up Wilcoxon signed rank tests with Bonferroni correction (significance level at .005) revealed that *neutral* state stimuli were rated as less intense than all other stimuli (*joy* (V = 812, *p* < .001, d = .383), *anger* (V = 562.5, *p* < .001, d = .494), *fear* (V = 1209, *p* = .007, d = .205), *sadness* (V = 1092, *p* = .001, d = .292). Besides, stimuli intended to express *anger* were rated as significantly higher in intensity than stimuli intended to express *fear* (V = 3207.5, *p* < .001, d = .294), and *sadness* (V = 2749.5, *p* = .048, d = .204). No other comparisons were significant (all ps > .142). See Fig. 3.

### Beauty ratings

**Figure 4:**
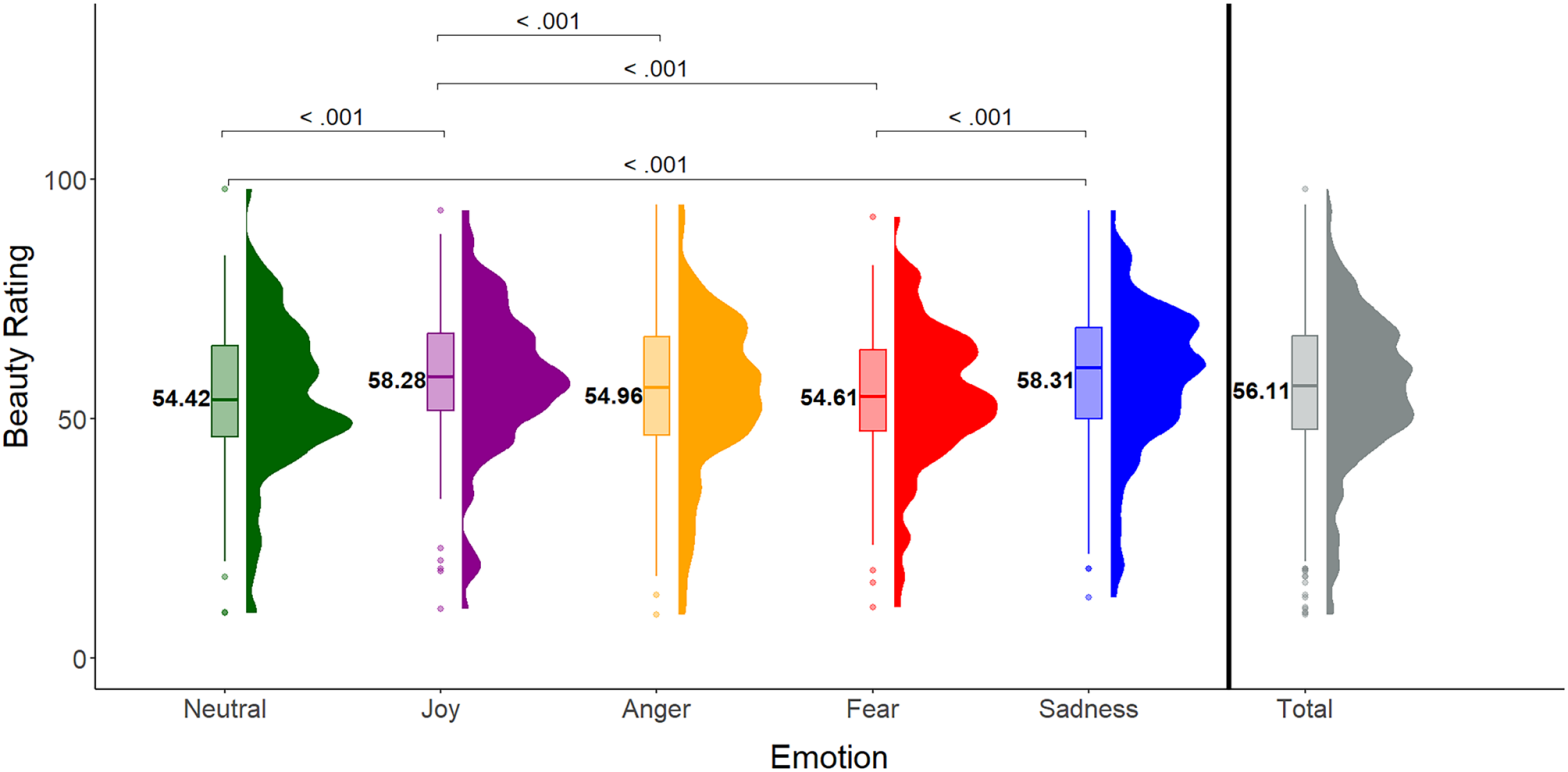
Average beauty ratings for 5 emotion categories. Mean and variability of Beauty ratings of dance movements, shown for all emotions as intended by the dancer. *p* values are Bonferroni-corrected.

A Friedman’s ANOVA showed a main effect of Intended Emotion on participants’ Beauty ratings (χ^2^(4) = 39.68, *p* < .001), suggesting that participants experienced the movements intended to express some emotions more beautiful than others. Follow-up Wilcoxon signed rank tests with Bonferroni correction (significance level at .005) revealed that stimuli intended to express *joy* were rated more beautiful than *anger* (V = 2901.5, *p* < .001, d = .208), *fearful* (V = 3260, *p* < .001, d = .241), and *neutral* state (V = 3071, *p* < .001, d = .241) stimuli. In addition, stimuli expressing *sadness* have higher Beauty ratings than *fearful* (V = 3088.5, *p* < .001, d = .241) and *neutral* state stimuli (V = 3152.5, *p* < .001, d = .242). See Fig. 4.

### Subjective emotion recognition

To explore how intensity and beauty ratings were distributed when using participants’ subjective emotion judgment (i.e., participants’ subjective perception of emotion, regardless of intended emotional expression by the dancer), the above analyses were repeated with the subjective emotion perception as grouping variable. No large differences between the two types of classifications were observed, as subjective perception and intended expression mostly overlapped. See supplementary materials section 2, for those analyses.

### Interindividual differences in emotion recognition and aesthetic judgement

We next explored how interindividual differences modulated emotion recognition, intensity ratings and aesthetic judgment. Only the personality trait conscientiousness predicted emotion recognition accuracy (conscientious individuals scored higher on the emotion recognition task). Intensity and beauty ratings were positively predicted by our overall engagement variable (“*how interesting did you find this task?*” 0 = not at all; 100 = very much; see “Methods” section). Beauty ratings were additionally predicted negatively by the personality trait negative emotionality. These regression analyses are set out in the supplementary materials (section 3). Regarding our variable dance experience, our sample had not been specifically recruited with this variable in mind. But because important previous research with dance professionals has shown links between dance experience and other neurocognitive processes^35,55–59^, dance experience data was collected as a means of experimental control. Participants’ average dance experience was very low (1.6 years; SD = 4.55), with many participants having none at all (81.1%, range = 0–30). As could be expected, this variable showed no effects neither on emotion recognition, nor on beauty or intensity ratings (see supplementary materials, section 3).

### Technical test

As a ‘technical test’ of the stimuli, we proceeded to inspect the emotion recognition rate for each stimulus. Of the 150 final stimuli, 139 had been recognized above chance level of 20%. We propose that any stimulus that was not recognized at least at 20% should not be used in subsequent experiments.

For stimulus selection in subsequent experiments, to leave sequences intact (i.e., where all five stimuli of a sequence have been recognized above chance level), we provide a table with all information about each sequence and each of the stimuli composing a sequence. We propose that only sequences where the intended emotional expression of all five stimuli have been recognized above chance level should be included in an experiment. A total of 22 sequences include stimuli that where all recognized above chance level, i.e., a total of 110 stimuli.

Table 3 shows the N = 150 stimuli of the stimuli set with their average Emotion Recognition Accuracy, Intensity Rating and Beauty Rating. Emotion Recognition Accuracies of stimuli were tested against chance level of 20% (100/5 = 20) by Boolean testing “Average Emotion Recognition Accuracy > 20?”. Krippendorff’s alpha was computed for each sequence to assess interrater reliability. See Table 3 for this data.

**Table 3:** Emotion recognition accuracies per stimulus and per sequence.

Stimulus name	Emotion encoded by dancer	Sequence number	Emotion decoded	Stimulus decoded above chance?	Intensity rating	Beauty rating	Sequence decoded above chance?	Included final set?	Krippendorff’s α of sequence
adagio01Sad	Sadness	1	76.67 (± 0.43)	Yes	71.87 (± 22.71)	68.23 (± 23.36)	No	No	.305
adagio01Angry	Anger	1	16.67 (± 0.38)	No	59.2 (± 19.26)	61.1 (± 21.22)	No	No	.305
adagio01Fear	Fear	1	60 (± 0.5)	Yes	62.87 (± 22.47)	57.27 (± 21.26)	No	No	.305
adagio01Happy	Joy	1	26.67 (± 0.45)	Yes	63.2 (± 20.82)	64.8 (± 25.18)	No	No	.305
adagio01Neutr	Neutral	1	53.33 (± 0.51)	Yes	57.43 (± 21.98)	57.77 (± 20.14)	No	No	.305
adagio02Fear	Fear	2	43.33 (± 0.5)	Yes	61.5 (± 20.61)	50.2 (± 23.48)	Yes (V = 15, * p* = .028)	Yes	.089
adagio02Happy	Joy	2	40 (± 0.5)	Yes	62.97 (± 16.3)	58.57 (± 25.09)	Yes (V = 15, * p* = .028)	Yes	.089
adagio02Neutr2	Neutral	2	53.33 (± 0.51)	Yes	51.97 (± 22.54)	53.6 (± 21.7)	Yes (V = 15, * p* = .028)	Yes	.089
adagio02Sad	Sadness	2	43.33 (± 0.5)	Yes	62.13 (± 20.59)	61.57 (± 21.92)	Yes (V = 15, * p* = .028)	Yes	.089
adagio02Angry	Anger	2	53.33 (± 0.51)	Yes	60.8 (± 20.93)	48.73 (± 21.66)	Yes (V = 15, *p* = .028)	Yes	.089
adagio03Fear	Fear	3	20 (± 0.41)	Yes	59.37 (± 18.86)	56.67 (± 21.89)	Yes (V = 10, *p* = .049)	Yes	.103
adagio03Happy3	Joy	3	66.67 (± 0.48)	Yes	57.73 (± 21.31)	57.73 (± 23.11)	Yes (V = 10, *p* = .049)	Yes	.103
adagio03Neutr	Neutral	3	56.67 (± 0.5)	Yes	57.53 (± 18.79)	56.8 (± 20)	Yes (V = 10, *p* = .049)	Yes	.103
adagio03Angry	Anger	3	43.33 (± 0.5)	Yes	62.6 (± 16.92)	60.97 (± 19.49)	Yes (V = 10, *p* = .049)	Yes	.103
adagio03Sad	Sadness	3	56.67 (± 0.5)	Yes	63.6 (± 17.87)	61.27 (± 20.64)	Yes (V = 10, *p* = .049)	Yes	.103
adagio04Fear	Fear	4	36.67 (± 0.48)	Yes	63.04 (± 21.13)	59.2 (± 20.87)	Yes (V = 15, *p* = .03)	Yes	.24
adagio04Happy	Joy	4	31.11 (± 0.47)	Yes	55.14 (± 18.86)	53.16 (± 21.32)	Yes (V = 15, *p* = .03)	Yes	.24
adagio04Neutr	Neutral	4	30 (± 0.46)	Yes	59.27 (± 18.67)	58.32 (± 21.13)	Yes (V = 15, *p* = .03)	Yes	.24
adagio04Angry	Anger	4	80 (± 0.4)	Yes	70.12 (± 20.26)	59.22 (± 21.81)	Yes (V = 15, *p* = .03)	Yes	.24
adagio04Sad	Sadness	4	77.78 (± 0.42)	Yes	65.44 (± 22.08)	58.31 (± 22.59)	Yes (V = 15, *p* = .03)	Yes	.24
adagio05Angry	Anger	5	53.33 (± 0.51)	Yes	58.47 (± 18.54)	53.97 (± 19.97)	Yes (V = 15, *p* = .03)	Yes	.296
adagio05Fear	Fear	5	26.67 (± 0.45)	Yes	63.07 (± 17.3)	55.87 (± 20.44)	Yes (V = 15, *p* = .03)	Yes	.296
adagio05Happy	Joy	5	33.33 (± 0.48)	Yes	51.87 (± 17.1)	49.07 (± 20.38)	Yes (V = 15, *p* = .03)	Yes	.296
adagio05Sad2	Sadness	5	63.33 (± 0.49)	Yes	68.67 (± 19.52)	52.57 (± 25.6)	Yes (V = 15, *p* = .03)	Yes	.296
adagio05Neutr	Neutral	5	46.67 (± 0.51)	Yes	59.17 (± 21.82)	45.93 (± 24.98)	Yes (V = 15, *p* = .03)	Yes	.296
adagio06Angry	Anger	6	33.33 (± 0.48)	Yes	64.6 (± 22.48)	63 (± 22.18)	Yes (V = 15, *p* = .03)	Yes	.105
adagio06Fear	Fear	6	30 (± 0.47)	Yes	55.93 (± 16.21)	57.57 (± 21.59)	Yes (V = 15, *p* = .03)	Yes	.105
adagio06Sad	Sadness	6	36.67 (± 0.49)	Yes	61.8 (± 17.31)	59.37 (± 22.3)	Yes (V = 15, *p* = .03)	Yes	.105
adagio06Happy	Joy	6	53.33 (± 0.51)	Yes	59.7 (± 15.75)	59.87 (± 23.56)	Yes (V = 15, *p* = .03)	Yes	.105
adagio06Neutr	Neutral	6	40 (± 0.5)	Yes	58.63 (± 18.51)	59.13 (± 22.86)	Yes (V = 15, *p* = .03)	Yes	.105
adagio07Angry	Anger	7	46.67 (± 0.51)	Yes	69.73 (± 19.21)	63.6 (± 25.3)	Yes (V = 15, *p* = .029)	Yes	.196
adagio07Fear	Fear	7	56.67 (± 0.5)	Yes	58 (± 19.42)	57.87 (± 22.03)	Yes (V = 15, *p* = .029)	Yes	.196
adagio07Neutr	Neutral	7	40 (± 0.5)	Yes	60.47 (± 18.09)	62.23 (± 24.3)	Yes (V = 15, *p* = .029)	Yes	.196
adagio07Sad	Sadness	7	60 (± 0.5)	Yes	65.67 (± 17.82)	64.1 (± 25.29)	Yes (V = 15, *p* = .029)	Yes	.196
adagio07Happy	Joy	7	60 (± 0.5)	Yes	65.57 (± 22.01)	61.23 (± 24.46)	Yes (V = 15, *p* = .029)	Yes	.196
adagio08Angry	Anger	8	43.33 (± 0.5)	Yes	67.57 (± 24.31)	59.87 (± 21.01)	Yes (V = 15, *p* = .029)	Yes	.057
adagio08Happy	Joy	8	50 (± 0.51)	Yes	58.1 (± 20.53)	58.23 (± 20.36)	Yes (V = 15, *p* = .029)	Yes	.057
adagio08Neutr	Neutral	8	43.33 (± 0.5)	Yes	53.97 (± 16.62)	60.53 (± 21.37)	Yes (V = 15, *p* = .029)	Yes	.057
adagio08Sad	Sadness	8	26.67 (± 0.45)	Yes	60.13 (± 18.95)	60.6 (± 21.19)	Yes (V = 15, *p* = .029)	Yes	.057
adagio08Fear	Fear	8	36.67 (± 0.49)	Yes	48.6 (± 21.88)	54.9 (± 24.2)	Yes (V = 15, *p* = .029)	Yes	.057
adagio09Happy	Joy	9	43.33 (± 0.5)	Yes	58.73 (± 21.78)	55.7 (± 21.8)	Yes (V = 10, *p* = .044)	Yes	.138
adagio09Neutr	Neutral	9	53.33 (± 0.51)	Yes	56.47 (± 20.63)	44.3 (± 23.6)	Yes (V = 10, *p* = .044)	Yes	.138
adagio09Sad	Sadness	9	20 (± 0.41)	Yes	55.23 (± 19.29)	52.4 (± 20.7)	Yes (V = 10, *p* = .044)	Yes	.138
adagio09Angry2	Anger	9	53.33 (± 0.51)	Yes	59.87 (± 22.4)	52.7 (± 22.62)	Yes (V = 10, *p* = .044)	Yes	.138
adagio09Fear	Fear	9	53.33 (± 0.51)	Yes	64.93 (± 17.01)	50.03 (± 21.84)	Yes (V = 10, *p* = .044)	Yes	.138
adagio10Angry	Anger	10	63.33 (± 0.49)	Yes	69.83 (± 20.06)	52.6 (± 26.8)	Yes (V = 15, *p* = .03)	Yes	.132
adagio10Fear	Fear	10	66.67 (± 0.48)	Yes	68.77 (± 23.44)	49.3 (± 24.62)	Yes (V = 15, *p* = .03)	Yes	.132
adagio10Happy	Joy	10	23.33 (± 0.43)	Yes	55.93 (± 16.41)	49.23 (± 20.64)	Yes (V = 15, *p* = .03)	Yes	.132
adagio10Neutr	Neutral	10	36.67 (± 0.49)	Yes	54.43 (± 20.43)	45.57 (± 22)	Yes (V = 15, *p* = .03)	Yes	.132
adagio10Sad	Sadness	10	73.33 (± 0.45)	Yes	69.33 (± 21.27)	54.73 (± 24.15)	Yes (V = 15, *p* = .03)	Yes	.132
adagio11Angry2	Anger	11	70 (± 0.47)	Yes	61.07 (± 25.74)	48.07 (± 21.37)	Yes (V = 15, *p* = .03)	Yes	.249
adagio11Fear2	Fear	11	53.33 (± 0.51)	Yes	61.23 (± 16.61)	47.4 (± 23.2)	Yes (V = 15, *p* = .03)	Yes	.249
adagio11Happy	Joy	11	76.67 (± 0.43)	Yes	69.13 (± 18.7)	58.77 (± 20.41)	Yes (V = 15, *p* = .03)	Yes	.249
adagio11Sad	Sadness	11	56.67 (± 0.5)	Yes	56.3 (± 22.49)	52.9 (± 17.28)	Yes (V = 15, *p* = .03)	Yes	.249
adagio11Neutr	Neutral	11	73.33 (± 0.45)	Yes	56.17 (± 24.49)	51.3 (± 26.25)	Yes (V = 15, *p* = .03)	Yes	.249
adagio12Fear	Fear	12	23.33 (± 0.43)	Yes	53.1 (± 21.16)	49.1 (± 19.07)	Yes (V = 15, *p* = .03)	Yes	.223
adagio12Happy	Joy	12	36.67 (± 0.49)	Yes	65.4 (± 22.73)	51.13 (± 24.34)	Yes (V = 15, *p* = .03)	Yes	.223
adagio12Neutr	Neutral	12	60 (± 0.5)	Yes	57.47 (± 24.41)	42 (± 20.06)	Yes (V = 15, *p* = .03)	Yes	.223
adagio12Angry	Anger	12	33.33 (± 0.48)	Yes	73.27 (± 19.57)	53.43 (± 24.13)	Yes (V = 15, *p* = .03)	Yes	.223
adagio12Sad	Sadness	12	73.33 (± 0.45)	Yes	67.13 (± 23.08)	52.3 (± 23.17)	Yes (V = 15, *p* = .03)	Yes	.223
adagio13Angry	Anger	13	63.33 (± 0.49)	Yes	59.07 (± 22.72)	53.93 (± 24.96)	Yes (V = 15, *p* = .027)	Yes	.098
adagio13Fear	Fear	13	56.67 (± 0.5)	Yes	55.5 (± 20.12)	55.3 (± 24.4)	Yes (V = 15, *p* = .027)	Yes	.098
adagio13Happy2	Joy	13	60 (± 0.5)	Yes	65.87 (± 20.09)	63.9 (± 18.02)	Yes (V = 15, *p* = .027)	Yes	.098
adagio13Sad	Sadness	13	56.67 (± 0.5)	Yes	58.4 (± 17.42)	61.4 (± 21.11)	Yes (V = 15, *p* = .027)	Yes	.098
adagio13Neutr	Neutral	13	56.67 (± 0.5)	Yes	53.93 (± 19.91)	58.7 (± 24.31)	Yes (V = 15, *p* = .027)	Yes	.098
adagio14Angry	Anger	14	53.33 (± 0.51)	Yes	63.83 (± 20.37)	48.9 (± 21.92)	No	No	.036
adagio14Fear2	Fear	14	13.33 (± 0.35)	No	52.6 (± 20.02)	47.67 (± 15.43)	No	No	.036
adagio14Sad	Sadness	14	16.67 (± 0.38)	No	54.17 (± 19.78)	49.5 (± 21.77)	No	No	.036
adagio14Happy	Joy	14	60.00 (± 0.5)	Yes	71.47 (± 19.13)	52.37 (± 20.56)	No	No	.036
adagio14Neutr	Neutral	14	60.00 (± 0.5)	Yes	56.2 (± 18.72)	46.77 (± 21.63)	No	No	.036
adagio15Fear	Fear	15	47.78 (± 0.5)	Yes	57.93 (± 21.97)	46.64 (± 22.61)	Yes (V = 15, *p* = .03)	Yes	.062
adagio15Happy	Joy	15	42.22 (± 0.5)	Yes	61.34 (± 19.83)	53.5 (± 21.21)	Yes (V = 15, *p* = .03)	Yes	.062
adagio15Angry	Anger	15	76.67 (± 0.43)	Yes	71.51 (± 19.63)	48.94 (± 23.84)	Yes (V = 15, *p* = .03)	Yes	.062
adagio15Neutr	Neutral	15	55.56 (± 0.5)	Yes	56.09 (± 20.17)	44.74 (± 22.94)	Yes (V = 15, *p* = .03)	Yes	.062
adagio15Sad	Sadness	15	40.00 (± 0.49)	Yes	57.84 (± 20.28)	49.3 (± 23.64)	Yes (V = 15, *p* = .03)	Yes	.062
adagio16Angry	Anger	16	53.33 (± 0.51)	Yes	57.9 (± 20.51)	48.37 (± 21.44)	Yes (V = 15, *p* = .029)	Yes	.421
adagio16Fear	Fear	16	50 (± 0.51)	Yes	55.3 (± 17.3)	55.1 (± 17.69)	Yes (V = 15, *p* = .029)	Yes	.421
adagio16Sad	Sadness	16	46.67 (± 0.51)	Yes	58.6 (± 18.34)	60.73 (± 14.73)	Yes (V = 15, *p* = .029)	Yes	.421
adagio16Happy	Joy	16	53.33 (± 0.51)	Yes	58.47 (± 19.09)	52.57 (± 20.74)	Yes (V = 15, *p* = .029)	Yes	.421
adagio16Neutr	Neutral	16	90 (± 0.31)	Yes	56.13 (± 21.58)	53.47 (± 20.66)	Yes (V = 15, *p* = .029)	Yes	.421
adagio17Angry2	Anger	17	30 (± 0.47)	Yes	57.47 (± 20.29)	46.57 (± 22.76)	Yes (V = 15, *p* = .03)	Yes	.426
adagio17Fear	Fear	17	23.33 (± 0.43)	Yes	58.03 (± 19.79)	53 (± 18.06)	Yes (V = 15, *p* = .03)	Yes	.426
adagio17Sad	Sadness	17	60 (± 0.5)	Yes	57.37 (± 21.49)	59.03 (± 17.75)	Yes (V = 15, *p* = .03)	Yes	.426
adagio17Happy	Joy	17	70 (± 0.47)	Yes	65.73 (± 20.3)	54.5 (± 21.15)	Yes (V = 15, *p* = .03)	Yes	.426
adagio17Neutr	Neutral	17	73.33 (± 0.45)	Yes	60.2 (± 19.45)	48.83 (± 19.43)	Yes (V = 15, *p* = .03)	Yes	.426
adagio18Angry	Anger	18	80 (± 0.41)	Yes	67.53 (± 21.44)	55.17 (± 22.71)	Yes (V = 15, *p* = .03)	Yes	.255
adagio18Neutr	Neutral	18	43.33 (± 0.5)	Yes	58.33 (± 17.24)	57.2 (± 18.81)	Yes (V = 15, *p* = .03)	Yes	.255
adagio18Sad	Sadness	18	83.33 (± 0.38)	Yes	69.8 (± 15.98)	64.37 (± 21.15)	Yes (V = 15, *p* = .03)	Yes	.255
adagio18Fear	Fear	18	40 (± 0.5)	Yes	65.1 (± 15.12)	53.1 (± 17.2)	Yes (V = 15, *p* = .03)	Yes	.255
adagio18Happy	Joy	18	23.33 (± 0.43)	Yes	56.4 (± 20.28)	63.23 (± 14.97)	Yes (V = 15, *p* = .03)	Yes	.255
adagio19Fear	Fear	19	13.33 (± 0.35)	No	49.77 (± 18.34)	54.97 (± 18.14)	No	No	.082
adagio19Happy	Joy	19	46.67 (± 0.51)	Yes	57.6 (± 16.32)	56.53 (± 19.99)	No	No	.082
adagio19Neutr	Neutral	19	56.67 (± 0.5)	Yes	55.07 (± 18.74)	53.57 (± 19.76)	No	No	.082
adagio19Sad	Sadness	19	36.67 (± 0.49)	Yes	55.87 (± 16.92)	56.7 (± 18.65)	No	No	.082
adagio19Angry	Anger	19	80 (± 0.41)	Yes	63.07 (± 20.91)	54.07 (± 20.77)	No	No	.082
adagio20Fear2	Fear	20	76.67 (± 0.43)	Yes	62.97 (± 17.59)	56.2 (± 19.99)	Yes (V = 15, *p* = .03)	Yes	.313
adagio20Happy	Joy	20	56.67 (± 0.5)	Yes	62.7 (± 16.16)	58 (± 20.2)	Yes (V = 15, *p* = .03)	Yes	.313
adagio20Neutr	Neutral	20	46.67 (± 0.51)	Yes	52.37 (± 18.57)	53.3 (± 18.77)	Yes (V = 15, *p* = .03)	Yes	.313
adagio20Angry	Anger	20	73.33 (± 0.45)	Yes	55.83 (± 19.44)	50.9 (± 21.93)	Yes (V = 15, *p* = .03)	Yes	.313
adagio20Sad	Sadness	20	33.33 (± 0.48)	Yes	53.07 (± 16.73)	53.67 (± 18.3)	Yes (V = 15, *p* = .03)	Yes	.313
adagio21Angry	Anger	21	63.33 (± 0.49)	Yes	56.07 (± 23.88)	52.8 (± 19.52)	Yes (V = 15, *p* = .03)	Yes	.131
adagio21Fear	Fear	21	30 (± 0.47)	Yes	58.33 (± 23.52)	58.2 (± 19.66)	Yes (V = 15, *p* = .03)	Yes	.131
adagio21Happy	Joy	21	40 (± 0.5)	Yes	54.77 (± 19.61)	58.07 (± 20.2)	Yes (V = 15, *p* = .03)	Yes	.131
adagio21Sad	Sadness	21	53.33 (± 0.51)	Yes	61.03 (± 15.56)	55.27 (± 21.39)	Yes (V = 15, *p* = .03)	Yes	.131
adagio21Neutr	Neutral	21	50 (± 0.51)	Yes	56.7 (± 20.11)	52.9 (± 22.08)	Yes (V = 15, *p* = .03)	Yes	.131
adagio22Fear	Fear	22	60 (± 0.5)	Yes	63.1 (± 22.69)	57.4 (± 19.35)	Yes (V = 15, *p* = .03)	Yes	.169
adagio22Happy	Joy	22	50 (± 0.51)	Yes	63.2 (± 17.71)	59.57 (± 18.78)	Yes (V = 15, *p* = .03)	Yes	.169
adagio22Neutr	Neutral	22	63.33 (± 0.49)	Yes	57.37 (± 18.56)	56.6 (± 19.92)	Yes (V = 15, *p* = .03)	Yes	.169
adagio22Angry	Anger	22	56.67 (± 0.5)	Yes	60.5 (± 19.42)	55.2 (± 19.36)	Yes (V = 15, *p* = .03)	Yes	.169
adagio22Sad	Sadness	22	46.67 (± 0.51)	Yes	55.47 (± 14.98)	56.33 (± 19.72)	Yes (V = 15, *p* = .03)	Yes	.169
adagio23Fear	Fear	23	50 (± 0.51)	Yes	54.97 (± 18.93)	55.5 (± 19.02)	Yes (V = 15, *p* = .03)	Yes	.154
adagio23Happy	Joy	23	53.33 (± 0.51)	Yes	57.03 (± 19.21)	56 (± 20.84)	Yes (V = 15, *p* = .03)	Yes	.154
adagio23Angry	Anger	23	63.33 (± 0.49)	Yes	62.07 (± 21.08)	56.73 (± 17.78)	Yes (V = 15, *p* = .03)	Yes	.154
adagio23Neutr	Neutral	23	23.33 (± 0.43)	Yes	52.4 (± 16.56)	52.5 (± 18.18)	Yes (V = 15, *p* = .03)	Yes	.154
adagio23Sad	Sadness	23	40 (± 0.5)	Yes	56.4 (± 19.55)	57.93 (± 18.31)	Yes (V = 15, *p* = .03)	Yes	.154
adagio24Angry2	Anger	24	23.33 (± 0.43)	Yes	64.27 (± 19.53)	55.23 (± 19.7)	Yes (V = 15, *p* = .03)	Yes	.416
adagio24Fear	Fear	24	33.33 (± 0.48)	Yes	54.4 (± 20.68)	50.1 (± 21.85)	Yes (V = 15, *p* = .03)	Yes	.416
adagio24Sad	Sadness	24	36.67 (± 0.49)	Yes	56.07 (± 19.36)	55.5 (± 18.96)	Yes (V = 15, *p* = .03)	Yes	.416
adagio24Happy	Joy	24	83.33 (± 0.38)	Yes	68.17 (± 17.1)	57.03 (± 15.35)	Yes (V = 15, *p* = .03)	Yes	.416
adagio24Neutr	Neutral	24	66.67 (± 0.48)	Yes	62.47 (± 19.25)	51.43 (± 23.22)	Yes (V = 15, *p* = .03)	Yes	.416
adagio25Angry2	Anger	25	86.67 (± 0.35)	Yes	69.07 (± 16.14)	55.17 (± 17.68)	No	No	.209
adagio25Fear	Fear	25	73.33 (± 0.45)	Yes	60.33 (± 19.62)	51.6 (± 21.52)	No	No	.209
adagio25Sad	Sadness	25	16.67 (± 0.38)	No	54.3 (± 19.87)	58.03 (± 19.58)	No	No	.209
adagio25Happy	Joy	25	60 (± 0.5)	Yes	61.43 (± 13.81)	55.73 (± 15.45)	No	No	.209
adagio25Neutr	Neutral	25	60 (± 0.5)	Yes	53.47 (± 19.81)	48.93 (± 20)	No	No	.209
ballet26Fear	Fear	26	7.78 (± 0.27)	No	59.91 (± 25.32)	50.13 (± 24.18)	No	No	.148
ballet26Angry	Anger	26	13.33 (± 0.34)	No	62.34 (± 23.46)	47.37 (± 25.23)	No	No	.148
ballet26Happy	Joy	26	15.56 (± 0.36)	No	57.61 (± 22.13)	56.74 (± 23.85)	No	No	.148
ballet26Neutr	Neutral	26	67.78 (± 0.47)	Yes	58.07 (± 25.52)	50.16 (± 25.42)	No	No	.148
ballet26Sad	Sadness	26	64.44 (± 0.48)	Yes	64.1 (± 23.81)	58.51 (± 25.65)	No	No	.148
ballet27Fear	Fear	27	20 (± 0.41)	Yes	66.5 (± 17.29)	61.8 (± 22.36)	No	No	.1
ballet27Angry	Anger	27	20 (± 0.41)	Yes	53.7 (± 24.46)	60.07 (± 25.49)	No	No	.1
ballet27Happy	Joy	27	16.67 (± 0.38)	No	60.3 (± 21.59)	66.03 (± 23.23)	No	No	.1
ballet27Neutr	Neutral	27	56.67 (± 0.5)	Yes	58.13 (± 24.84)	66.3 (± 25.1)	No	No	.1
ballet27Sad	Sadness	27	36.67 (± 0.49)	Yes	60.07 (± 20.38)	61.9 (± 27.74)	No	No	.1
ballet28Angry	Anger	28	30 (± 0.47)	Yes	56.7 (± 17.58)	62.03 (± 21.44)	Yes (V = 15, *p* = .029)	Yes	.113
ballet28Sad	Sadness	28	43.33 (± 0.5)	Yes	61.17 (± 21.26)	68.4 (± 18.85)	Yes (V = 15, *p* = .029)	Yes	.113
ballet28Fear	Fear	28	50 (± 0.51)	Yes	60.57 (± 21.13)	59.6 (± 23.46)	Yes (V = 15, *p* = .029)	Yes	.113
ballet28Happy	Joy	28	66.67 (± 0.48)	Yes	65.57 (± 16.4)	68 (± 24.87)	Yes (V = 15, *p* = .029)	Yes	.113
ballet28Neutr	Neutral	28	50 (± 0.51)	Yes	52.83 (± 21.38)	65.5 (± 18.79)	Yes (V = 15, *p* = .029)	Yes	.113
ballet29Sad	Sadness	29	40 (± 0.5)	Yes	62 (± 16.86)	67.5 (± 20.23)	No	No	.072
ballet29Angry2	Anger	29	10 (± 0.31)	No	63.77 (± 24.94)	66.1 (± 22.07)	No	No	.072
ballet29Fear	Fear	29	33.33 (± 0.48)	Yes	56.47 (± 24.08)	64.53 (± 21.66)	No	No	.072
ballet29Happy	Joy	29	86.67 (± 0.35)	Yes	70.8 (± 19.16)	71.07 (± 23.57)	No	No	.072
ballet29Neutr2	Neutral	29	33.33 (± 0.48)	Yes	56.83 (± 20.88)	60.67 (± 22.32)	No	No	.072
ballet30Fear	Fear	30	16.67 (± 0.38)	No	55 (± 21.4)	63.47 (± 19.75)	No	No	.063
ballet30Happy3	Joy	30	70 (± 0.47)	Yes	71.77 (± 17.41)	70.87 (± 20.69)	No	No	.063
ballet30Neutr	Neutral	30	60 (± 0.5)	Yes	53.77 (± 22.37)	68.53 (± 20.56)	No	No	.063
ballet30Sad	Sadness	30	33.33 (± 0.48)	Yes	55.53 (± 24.65)	65.2 (± 21.49)	No	No	.063
ballet30Angry	Anger	30	26.67 (± 0.45)	Yes	57.4 (± 24.45)	61.07 (± 21.45)	No	No	.063

All N = 150 stimuli of the stimuli set with their average Emotion Recognition Accuracy, Intensity Rating and Beauty Rating. Emotion Recognition Accuracies of stimuli were tested against chance level of 20% (100/5 = 20) by Boolean testing “Average Emotion Recognition Accuracy > 20?”. Krippendorff’s alpha was computed for each sequence to assess interrater reliability.

## Discussion

We created an emotional dance movement stimuli-set for emotion psychology and related disciplines. It contains 30 dance sequences performed five times each, with five different intended emotional expressivities at each repetition (joy, anger, fear, sadness, and a neutral state), i.e., a total of 150 stimuli. Emotion recognition for all five emotion categories as intended by the dancer were recognized above chance level (chance: 20%; joy: 45%, anger: 48%, fear: 37%, sadness: 50%, neutral state: 51%). Fear had significantly lower emotion recognition rates than the rest of the emotion categories, but was still above chance. This finding is in accordance with previous literature in which the difficulty to recognize fear from full-body movements has been reported ^44^. One-hundred-thirty-nine of the 150 stimuli were recognized above chance level. Respecting sequence membership, data showed that all five stimuli of a total of 22 sequences were recognized above chance level. This means that for leaving sequence-membership intact, a set of 110 stimuli (22 sequences × 5 emotions) can be used from this emotional dance movement stimuli set.

Importantly, as a manipulation check, the neutral state stimuli (neutral expressivity), were rated as less intense than all other emotions, confirming that these neutral state stimuli were *less* emotionally expressive in intensity, as had been intended by the dancer. Thus, this category can be used as a control condition. We found no difference between anger and joy in terms of intensity, as has been reported before. Anger was rated as more intense than the stimuli intended to express sadness and fear, and joy was rated as more intense than neutral (joy = anger; joy > neutral; anger > fear/sadness > neutral).

Regarding our conjecture about *implicit* emotion recognition via aesthetic judgment, we found that participants’ aesthetic judgment (beauty ratings) was indeed sensitive to the intended emotion by the dancer. Stimuli expressive of joy and sadness received the highest beauty ratings, fear and neutral expressivity received the lowest (joy > anger > fear > neutral, and sad > fear > neutral). Interestingly, the high arousal emotions anger and joy were rated as equally intense, but participants’ beauty ratings differed between the two emotions, with joyful movements being rated as more beautiful, than angry movements. On the other hand, low-intensity stimuli expressing sadness were rated as more beautiful, than other low-intensity stimuli including neutral state and fearful stimuli. These results suggest that aesthetic judgment could indeed be conceptualized as a type of implicit emotion recognition task.

Interindividual difference measures of personality and aesthetic responsiveness did not significantly predict emotion recognition accuracy, except for conscientiousness that predicted higher emotion recognition accuracy. Our engagement measure ‘interest in task’ predicted intensity ratings and beauty judgments, while beauty judgments were also negatively predicted by the personality trait negative emotionality.

## Overall discussion and conclusion

It has long been argued that accurate emotion recognition from conspecifics confers an evolutionarily adaptive advantage to the individual^22,45,60,61^, yet results remain mixed^62,63^. Importantly, while there are different channels of emotional expressivity (face, voice, and the body), few validated full-*body* stimuli sets are available to test for emotion recognition effects and their possible links to broader cognitive function. This is an important pitfall, especially, as some research suggests that a high recognition accuracy, specifically, for *bodily* expressions of emotion (as opposed to facial expressions of emotions) could be associated with *negative* psychosocial outcomes^2,10^.

Therefore, we here propose dance movements as stimuli for emotion science, to answer a range of questions about human full-body emotion perception^13,14,64–68^. Echoing this, we created and validated a set of 150 full-body dance movement stimuli for research in emotion psychology, affective neuroscience and empirical aesthetics. We provide emotion recognition rates, intensity ratings and aesthetic judgment values for each stimulus, and have demonstrated emotion recognition rates above chance for 139 of the 150 stimuli. We also provide first data to suggest that aesthetic judgment to this carefully controlled stimuli-set could serve as a useful implicit emotion recognition task.

## Methods

Ethical approval for the experiment was in place through the Umbrella Ethics approved by the Ethics Council of the Max Planck Society (Nr. 2017_12). Informed consent was obtained from all participants and/or their legal guardians. The informed consent was given online through a tick-box system. All methods were performed in accordance with the relevant guidelines and regulations.

### Participants: the dancer

One professional dancer from the Dresden Frankfurt Dance Company, Germany, collaborated and was remunerated as model for all stimuli. The dancer was a professional dancer trained in classical ballet technique, but working in a professional dance company where Western contemporary dance was the main mode of expression.

### Participants: the observers

Participant characteristics of the 90 participants are set out in Table 4.

**Table 4:** Sociodemographic characteristics of participants.

Variable	Full sample				
	*n*	%	*M*	*SD*	Range
Gender					
Female	56	62.2			
Male	34	37.7			
Other	0	0			
Missing	0	0			
Age			33.19	12.26	18–66
Education					
High school diploma	31	34.4			
Bachelor’s degree	40	44.4			
Master’s degree	10	11.1			
Other	9	10			
Years of dance experience			1.66	4.55	0–30

*N* = 90 participants were on average 33.19 years old (*SD* = 12.26, range: 18–66). Participants had dance experience of 1.66 years on average (*SD* = 4.55, range: 0–30).

The sample size was determined as follows. The final stimuli number (n = 173 including duplicates; see “Stimuli” section) would have been too many stimuli to rate for participants in one experiment. Therefore, stimuli were divided into 3 sets. Each set was rated by a different group of participants, and we planned to compare these three groups in terms of their ratings to 15 shared stimuli to evaluate interrater reliability. Sample size was determined separately for these groups, using G*Power 3.1^69^. Choosing the threshold of a large effect size of *d* = .80^70^, our sample size calculation for independent samples t-test (effect size = .80; alpha = .05; power = .90) suggested a sample size of 28 per group. We tested 30 participants per group to ensure full randomization (30 is divisible by 5 emotions, 28 is not).

### Materials

#### Stimuli

We used N = 173 video clips of 6 s length of a white silhouette dancer on black background. Stimuli contained no facial information, no costume, nor music. Each clip was faded in and out.

A dancer choreographed 30 sequences of dance movements. Of the 30 sequences, five were Western classical ballet, the rest were Western contemporary dance. The length was 8 counts in dance theory, ~ 8 s. The dancer performed each sequence five times each with different emotional expressivity at each repetition; joy, fear, anger, sadness and neutral state. A total of 173 stimuli were recorded instead of 150 (30 sequences × 5 emotions = 150 stimuli): When the dancer wasn’t satisfied with her performance of a sequence, she asked to repeat it. Therefore, some of the stimuli were repeated. All 173 stimuli were included in the experiment to be able to select the “best” stimuli based on emotion recognition data. The 23 additional takes were deleted before analysis, by selecting the stimulus with the highest emotion recognition rate among duplicates. See Fig. 1 for an illustration of the stimuli creation process and a sample stimulus.

#### Questionnaires

Participants provided demographic information and interindividual difference measures were collected. First, the personality measure Big Five Inventory Short version (BFI-S)^71,72^ that contains five subscales, namely Agreeableness, Conscientiousness, Extraversion, Negative Emotionality and Open-mindedness. Second, the Aesthetic Responsivity Assessment (AReA)^73^ that screens for sensitivity and engagement with the arts. It contains 14 items (answers were given on a 5-point Likert scale between 0 (never) and 4 (very often)) that split into three first-order factors: Aesthetic Appreciation (AA; how much an individual appreciates different types of art, like poetry, paintings, music, dance), Intense Aesthetic Experience (IAE; an individual’s propensity to experience a subset of more intense aesthetic experiences like being moved, awe or the sublime), and Creative Behaviour (CB; an individual’s propensity to actively engage in creative processes like writing, painting, music making or dancing).

Participants had an average of 1.6 years (SD = 4.55) of dance experience, with many participants having no dance experience at all (81%, range 0 – 30).

#### Attention and engagement checks

A series of attention checks controlled for engagement: On two trials of the questionnaires, participants were asked “*please press the central circle*” and non-compliance lead to exclusion. On two of the emotion recognition trials, cartoon videos were shown with very obvious emotional expressions (Sponge Bob crying a river of tears; correct response: sad; and Mikey Mouse’s head turning red and exploding; correct response: angry). Participants who rated these incorrectly were excluded. Finally, a question was added after the emotion and aesthetics rating tasks, “Did the videos play alright?*”* (0 = not at all; 5 = yes, all good). Participants who rated 3 or less were excluded.

A final question in the experiment asked participants how interesting they found the task they had just participated in. This is because previous research suggests that the personal interest in the task modulates task engagement and quality of responses^32,43,74^. We included this variable in the regression models.

### Procedure

See Fig. 1 for the stimuli creation procedure.

To obtain normative values, the N = 173 video clips were divided into three sets and presented to three separate groups of 30 participants. Three randomly chosen sequences (= 15 stimuli) were included in all three sets for interrater reliability assessments between the three groups. Including the three ‘shared’ sequences, the resulting three stimuli sets were as follows: Set 1 included only the ballet sequences (seven sequences) and consisted of 39 stimuli (including 4 additional takes). Set 2 included contemporary dance sequences (15 sequences) and consisted of 84 stimuli (including nine additional takes), and Set 3 included contemporary dance sequences (14 sequences) and consisted of 80 stimuli (including 10 additional takes).

The experiment was set up on Limesurvey®, where participants were also asked to read an information sheet and sign the consent form. Participants signed up for the rating experiment online via the Prolific© platform. The experiment began with the demographics questionnaire, followed by the emotion recognition task including beauty and intensity ratings, followed by the remaining questionnaires.

On each trial, participants were shown one dance video stimulus (randomized presentation), and then a forced-choice paradigm was used where participants were asked to select one emotion the dancer was intending to express (joy, anger, fear, sadness or neutral state). It was not possible to repeat the video after it had played one time. Two slider questions from 0 (not at all) to 100 (very much) probed for perceived intensity of the emotional expression and beauty of the movement (i.e., “How intensely was the emotion expressed?”/“How beautiful did you find the movement?”)*.* “Intensity” was added as a proxy measure of “power” commonly used in emotion research. However, research participants find it difficult to rate “power” and we opted for “intensity” instead.

For a qualitative assessment, we added an open question, where participants were invited to indicate any other emotions that they perceived in the movement, by writing the emotion in a box (this data is not analysed in this manuscript). Participants were debriefed about the objectives of the experiment at the end.

## Supplementary Information

Supplementary Information.

## Data Availability

The stimuli set, normative values and code are available for download here: https://osf.io/uecg9/?view_only=e5a5661b89104701aca750101325d30f and a short video about the stimuli creation is available here: https://www.youtube.com/watch?v=Eij40jtw8WE.
