# Supplementary material for: A 5-emotions stimuli set for emotion perception research with full-body dance movements
Source: Sci Rep. 2023 May 30;13:8757. doi: 10.1038/s41598-023-33656-4 (PMC10229576; doi:10.1038/s41598-023-33656-4)
Supplement: Supplementary file 1 — Supplementary Information. [file 41598_2023_33656_MOESM1_ESM.docx]

**SUPPLEMENTARY MATERIAL FOR:**

**A 5-Emotions Stimuli Set For Emotion Perception Research With Full-Body Dance Movements**

Julia F. Christensen^1^, Laura Bruhn^1^, Eva-Madeleine Schmidt^1^, Nasimeh Bahmanian^1,2^, Sina H.N. Yazdi^3^, Fahima Farahi^3^, Luisa Sancho-Escanero^4^, Winfried Menninghaus^1^

^1^Max Planck Institute for Empirical Aesthetics, Frankfurt/M, Germany

^2^Department of Modern Philology, Goethe University, Frankfurt/M, Germany

^3^WiseShot, Porto, Portugal

^4^Pfalztheater Kaiserslautern Dance Company, Germany

**Index Page**

1. Preliminary data analyses P. 2
2. Subjective emotion judgments and beauty and intensity ratings P. 3
3. Interindividual differences in Emotion Recognition and aesthetic judgement P. 5
   1. Regression for Emotion Recognition Accuracy P. 5
   2. Regression for Beauty Ratings P. 6
   3. Regression for Intensity Ratings P. 7
4. **Preliminary data analyses**

Fifteen videos of the full dance movement library had been included in all three online experiments. To confirm that emotion recognition rates between the three sets of stimuli were equivalent, we performed comparative analyses.

A Kruskal-Wallis test was used to determine whether there was a difference between the three groups in terms of their Emotion Recognition rates to stimuli. Emotion Recognition rates did not differ significantly between groups (H(2) = .179, p = .914). The same was true for the recognition rate of each emotion specifically, i.e., for neutral state stimuli (H(2) = 1.175, p = .556), for joyful stimuli (H(2) = .627, p = .731), for angry stimuli (H(2) = .433, p = .805), for fearful stimuli (H(2) = 1.322, p = .516), and for sad stimuli (H(2) = 1.867, p = .393).

We repeated this analysis to test whether Intensity ratings differed significantly between groups. Again, no general effect was found (H(2) = .341, p = .843). Stimuli were rated equivalently in all three online experiments in terms of intensity; and the same was true when testing the Intensity ratings of each emotion specifically (neutral state: H(2) = 1.689, p = .43; joyful: H(2) = .089, p = .957; angry: H(2) = .622, p = .733; fearful: H(2) = .622, p = .733; sad: H(2) = 3.289, p = .193).

Finally, the analysis was repeated to test whether Beauty ratings differed between groups. A general effect was found (H(2) = 7.74, p = .021), and post-hoc tests showed that groups 1 and 3 differed significantly (*difference* = 12.6, significance level = .05). We continued to test Beauty ratings specifically for each emotion, but found no effect (neutral state stimuli: H(2) = 1.067, p = .587, joyful stimuli: H(2) = 5.6, p = .061, angry stimuli: H(2) = 2.22, p = .329, fearful stimuli: H(2) = 1.156, p = .561, sad stimuli: H(2) = 3.2, p = .202).

Despite the significant difference in beauty ratings (which could be due to the different type of dance (ballet vs contemporary dance, an effect also found in Christensen et al., 2018, 2021), we consider the ratings of participants from all three sets to be equivalent and that they can be analysed together. Additionally, interrater reliability was high across the three Sets for Emotion Recognition (Krippendorff’s α = .833), and moderate for Intensity ratings (Krippendorff’s α = .574) and for Beauty ratings (Krippendorff’s α = .537).

1. **Subjective emotion judgments and beauty and intensity ratings**

We proceeded to explore participants’ own emotion judgments of the stimuli, i.e., independent of the intended emotion of the dancer.

A Friedman’s ANOVA showed a main effect of Subjective Emotion Judgment of participants’ Intensity ratings (χ2(4) = 59.995, p < .001), suggesting that participants experienced some emotions as more intense than others. Follow-up Wilcoxon signed rank tests with Bonferroni correction (significance level at .001) revealed that stimuli experienced as *neutral* were rated less intense than *joyful* (V = 1095, p = .001, d = .656), *angry* (V = 1087, p = .001, d = .587), and *sad* (V = 1316, p = .033, d = .49) stimuli. See figure S1.

**Figure S1**

*Intensity Ratings for Subjective Emotion Judgment Classification*


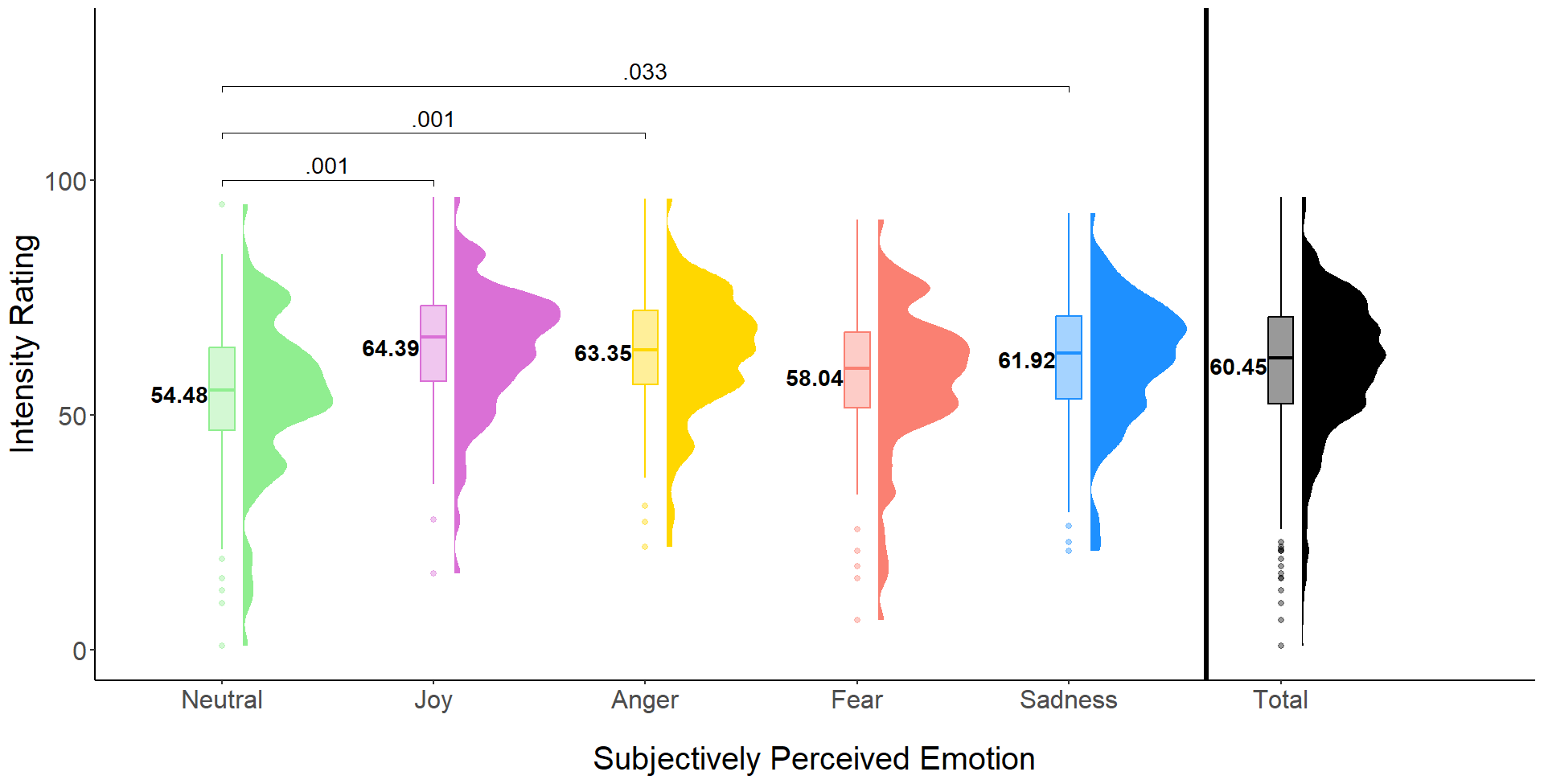


*Note*: Mean and variability of Intensity ratings of dance movements, shown for all emotions as perceived by the observer (Subjective Emotion Judgment). P-values are Bonferroni-corrected.

A Friedman’s ANOVA showed a main effect of Subjective Emotion Judgment of participants’ Beauty ratings (χ2(4) = 126.12, p < .001), suggesting that participants experienced some emotions more beautiful than others. Follow-up Wilcoxon signed rank tests with Bonferroni correction (significance level at .005) revealed that stimuli experienced as *joyful* were rated more beautiful than *angry* (V = 2899.5, p = .006, d = .556) and *neutral* (V = 3200, p < .001, d = .699) stimuli. Also, stimuli experienced as *sad* have higher Beauty ratings than *angry* (V = 2789, p = .029, d = .456) and *neutral* (V = 2965, p = .002, d = .599) stimuli. See figure S2.

**Figure S2**

*Beauty Ratings for Subjective Emotion Judgment Classification*


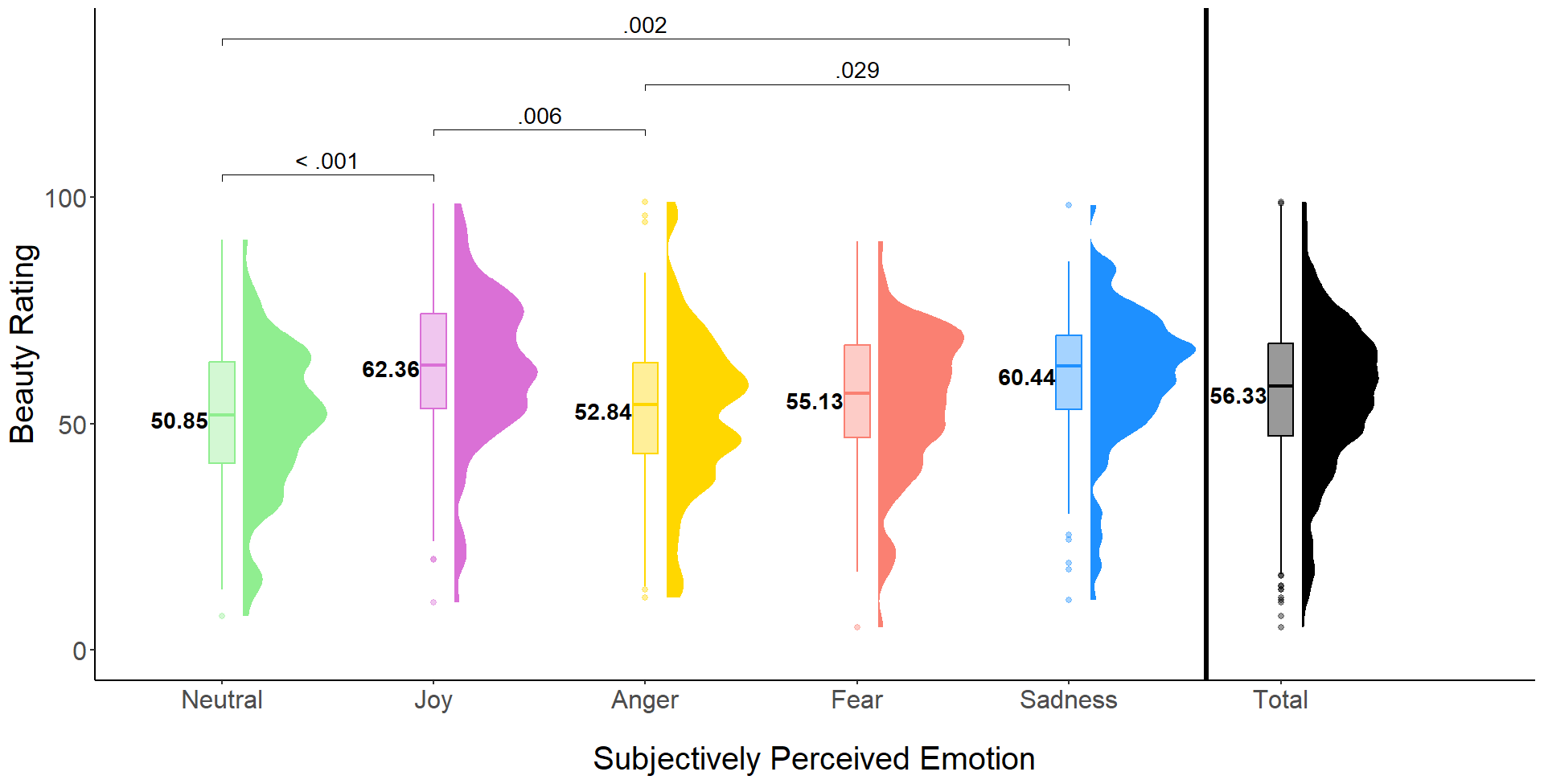


*Note*: Mean and variability of Beauty ratings of dance movements, shown for all emotions as perceived by the observer. P-values are Bonferroni-corrected.

1. **Interindividual differences in Emotion Recognition and aesthetic judgement**
   1. **Regression for Emotion Recognition Accuracy**

A multiple linear regression was performed with the dependent variable Emotion Recognition Accuracy and as predictors the Aesthetic Responsiveness Assessment (AReA) factors (Aesthetic Appreciation, Creative Engagement, and Intense Aesthetic Experience), the Big Five Inventory (BFI) personality factors (Agreeableness, Conscientiousness, Extraversion, Negative Emotionality, Open-Mindedness), Years of Dance Experience, Interest in Task (Likert rating; 0 = not interesting at all; 5 = very interesting), and Gender (0 = male, 1 = female).

The regression was not significant, *F*(9, 80) = 1.826, *p* = .076, with an adjusted R^2^ of .077, meaning that 7.7% of the variance was explained by this model. Significant predictors were BFI Conscientiousness (*t =* 2.028, β = .252, *p <* .046). No other predictors modulate the Emotion Recognition Accuracy. See table S1 for this regression.

**Table S1**

*Regression model prediciting Emotion Recognition Accuracy*

| Variable |  |  |  |  |
| --- | --- | --- | --- | --- |
|  | *B* | β | *SE* | *p* |
| Constant | 23.66 |  | 11.64 | .046* |
| AReA Total | .21 | .234 | .12 | .089 |
| Agreeableness | -1.01 | -.078 | 1.49 | .498 |
| Conscientiousness | 2.9 | .252 | 1.42 | .046* |
| Extraversion | .94 | .085 | 1.45 | .523 |
| Negative Emotionality | 1.64 | .173 | 1.39 | .241 |
| Open-mindedness | 1.54 | .106 | 1.89 | .416 |
| Years of Dance Experience | .19 | .097 | .22 | .373 |
| Interest in Task | -.182 | -.023 | .92 | .052^+^ |
| Gender | 2.91 | .155 | 2.07 | .165 |

*Note*: Coefficients of the model are adjusted R^2^ = .077, *F*(9, 80) = 1.826, *p* = .076, *N* = 90. Predictors: Personality Traits (Agreeableness, Conscientiousness, Extraversion, Negative Emotionality, and Open-mindedness), Aesthetic Responsiveness (AReA), years of dance experience, interest in task and gender. Dependent variable: Emotion Recognition Accuracy. * > .05; ^+^ = marginal effect.

- 1. **Regression for Beauty Ratings**

A multiple linear regression was performed with the dependent variable Beauty Ratings and as before. A significant regression equation was found, *F(*9, 80) = 4.711, *p <* .001, with an adjusted R^2^ of .273, meaning that 27.3% of the variance was explained by this model. Significant predictors were BFI Negative Emotionality (*t =* -2.408, β = - .313, *p* = .018) and Interest in Task (*t =* 3.708, β = .378, *p <* .001). No other predictors modulated Beauty Ratings. See table S2 for this regression.

**Table S2**

*Regression Model Predicting Beauty Ratings*

| Variable |  |  |  |  |
| --- | --- | --- | --- | --- |
|  | *B* | β | *SE* | *p* |
| Constant | 39.755 |  | 16.857 | .021* |
| Aesthetic Responsiveness Total | 0.013 | .009 | .176 | .941 |
| Agreeableness | 1.451 | .069 | 2.158 | .503 |
| Conscientiousness | -.696 | -.037 | 2.069 | .737 |
| Extraversion | -.286 | -.016 | 2.11 | .893 |
| Negative Emotionality | -.4.848 | -.313 | 2.014 | .018* |
| Open-mindedness | 2.337 | .098 | 2.73 | .395 |
| Years of Dance Experience | -.408 | -.125 | .315 | .198 |
| Interest in Task | 4.95 | .378 | 1.335 | .001** |
| Gender | 5.962 | .186 | 3.004 | .062 |

Note. Coefficients of the model are adjusted R^2^ = .35, *F(*9, 80) = 4.711, *p <* .001, *N* = 90. Predictors: Personality Traits (Agreeableness, Conscientiousness, Extraversion, Negative Emotionality, and Open-mindedness), Aesthetic Responsiveness (AReA), years of dance experience, interest in task and gender. Dependent variable: Beauty ratings.

- 1. **Regression for Intensity Ratings**

A multiple linear regression was performed with the dependent variable Intensity Ratings and predictors as before. A trend towards a significant regression was found, *F(*9, 80) = 1.963, *p* = .055, with an adjusted R^2^ of .089, meaning that 8.9% of the variance was explained by this model. A significant predictor was Interest in Task (*t =* 2.897, β = .33, *p* = .005). No other predictors modulated the Intensity Ratings. See table S3 for this regression.

**Table S3**

*Regression Model predicting Intensity Ratings*

| Variable |  |  |  |  |
| --- | --- | --- | --- | --- |
|  | *B* | β | *SE* | *p* |
| Constant | 54.409 |  | 15.698 | < .001*** |
| Aesthetic Responsiveness Total | -.07 | -.057 | .164 | .672 |
| Agreeableness | -1.631 | -.093 | 2.01 | .42 |
| Conscientiousness | -.994 | -.064 | 1.926 | .607 |
| Extraversion | 1.407 | .095 | 1.965 | .476 |
| Negative Emotionality | -2.473 | -.192 | 1.875 | .191 |
| Open-mindedness | 2.468 | .125 | 2.542 | .335 |
| Years of Dance Experience | -.063 | -.023 | .293 | .829 |
| Interest in Task | 3.601 | .33 | 1.243 | .005** |
| Gender | .738 | .029 | 2.797 | .792 |

*Note*: Coefficients of the model are R^2^ = .089, *F(*9, 80) = 1.963, *p* = .055, *N* = 90. We examined the effect of multiple interindividual difference measures as predictors: The Big Five personality dimensions (Agreeableness, Conscientiousness, Extraversion, Negative Emotionality, and Open-mindedness), AReA (the Aesthetic Responsiveness Assessment), years of dance experience, interest in task, and gender on how intense participants perceived the dance movement stimuli.
